# Inappropriate surgery in a patient with misdiagnosed Robert’s uterus

**DOI:** 10.1186/s12905-021-01404-3

**Published:** 2021-07-03

**Authors:** Iori Kisu, Kanako Nakamura, Tetsuro Shiraishi, Tomoko Iijima, Moito Iijima, Kiyoko Matsuda, Nobumaru Hirao

**Affiliations:** 1 Department of Obstetrics and Gynecology, Federation of National Public Service Personnel Mutual Aid Associations, Tachikawa Hospital, 4-2-22 Nishiki-cho, Tachikawa-shi, Tokyo 1908531 Japan; 2 Department of Obstetrics and Gynecology, Keio University School of Medicine, 35 Shinanomachi, Shinjuku-ku, Tokyo, 1608582 Japan

**Keywords:** Robert’s uterus, Mullerian anomaly, Septate uterus, Dysmenorrhea, Rudimentary horn, Unicornuate uterus, Hematometra

## Abstract

**Background:**

Robert’s uterus is a rare Mullerian anomaly, which can be described as an asymmetric, septate uterus with a non-communicating hemicavity. Herein, we present the case of a misdiagnosed Robert’s uterus, resulting in an invasive and disadvantageous surgery.

**Case presentation:**

A 16-year-old woman was referred to our department because of dysmenorrhea and suspicion of uterine malformation. We misdiagnosed Robert’s uterus as a unicornuate uterus with a non-communicating rudimentary horn and hematometra, and performed laparoscopic hemi-hysterectomy. Although the patient’s symptoms were relieved, our surgical procedure left the lateral uterine wall weak, making the patient’s uterus susceptible to uterine rupture in any future pregnancy.

**Conclusions:**

Although the early diagnosis of Robert’s uterus is challenging, it is important in order to determine appropriate surgical interventions and management for maintaining the quality of life and ensuring safety in future pregnancies.

## Background

Robert’s uterus is an extremely rare Mullerian anomaly associated with a variant of septate uterus with a non-communicating hemicavity and hematometra, which was first reported by Robert in 1970 [1]. Only a few cases of the condition have been reported in the literature to date. It is characterized by an asymmetrical, septate uterus with the obstruction of a one-sided cavity and formation of hematometra. The other cavity communicates normally with the single cervix. Retention of menstrual blood associated with the condition can cause periodical abdominal pain. As Robert’s uterus is a very rare disease, it can be easily misdiagnosed or mistreated. Herein, we report a case of Robert’s uterus, which was misdiagnosed as a unicornuate uterus with a non-communicating rudimentary horn. Although dysmenorrhea was resolved with our treatment, the misdiagnosis resulted in a disadvantageous surgery.

## Case presentation

A 16-year-old woman with a chief complaint of lower abdominal pain presented to another hospital and was suspected to suffer from a uterine malformation; she had a bicornuate uterus with left intrauterine hematoma. Hence, she was subsequently referred to our hospital for specialized treatment. The patient was suffering from severe lower abdominal pain that required absence from school every month since she was 15 years old. She had attained menarche at the age of 13 years, and her menstrual cycles had been regular since then.

**Fig. 1:**
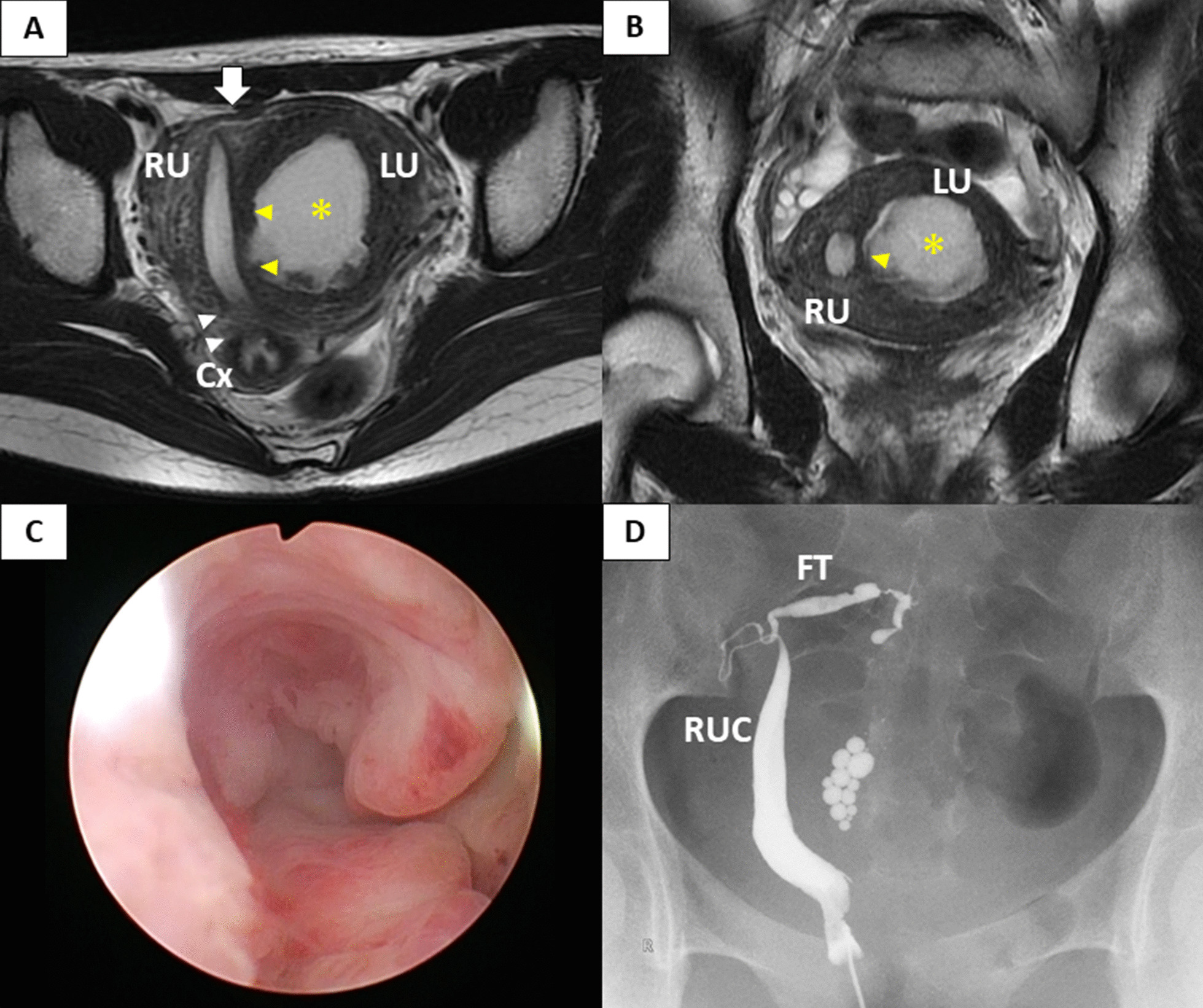
Pre-operative findings. MRI scan in axial (**a**) and coronal (**b**) planes showing the right uterus and the left uterus with a 5 cm-sized hematometra (*) in the uterine cavity. An asymmetric uterine septum is found between the left and right uterine cavity, which are not communicating (yellow triangles). One cervix is confirmed and connected to the right uterus body (white triangles). The left and right uterine fundus are clearly not divided with a normal uterine fundal contour (white arrow). Hysteroscopy in the right uterus revealed a simple small cavity, not communicating with the left uterus (**c**). Hysterosalpingography also showed no traffic to the left uterine cavity and the compressed shape of the right uterine hemi-cavity with fallopian tube patency (**d**). *RU* right uterus, *LU* left uterus, *RUC* right uterine cavity, *FT* fallopian tube

Speculum examination revealed a single cervix with no vaginal septum. Transvaginal ultrasonography (US) revealed a normal form of the right and left uteri, which contained blood consistent with hematometra. Her bilateral kidneys were present and normal on transabdominal US. Pelvic magnetic resonance imaging (MRI) revealed the right uterus and the left uterus with a 5 cm-sized hematometra in the uterine cavity, and confirmed an asymmetrical uterine septum between the left and right endometrial cavities. The left and right uterine fundus were clearly not separated with a normal uterine fundal contour (Fig. 1a, b). Hysteroscopy identified the ostium of the right fallopian tube, but did not confirm aby communication with the left uterus (Fig. 1c). Hysterosalpingography also showed no traffic to the left uterine cavity, only 3 mL of contrast medium filling the right uterine hemi-cavity with a compressed shape (Fig. 1d). Based on the above findings, a misdiagnosis of a right unicornuate uterus with a non-communicating left rudimentary horn was made, and laparoscopic surgery was performed.

**Fig. 2:**
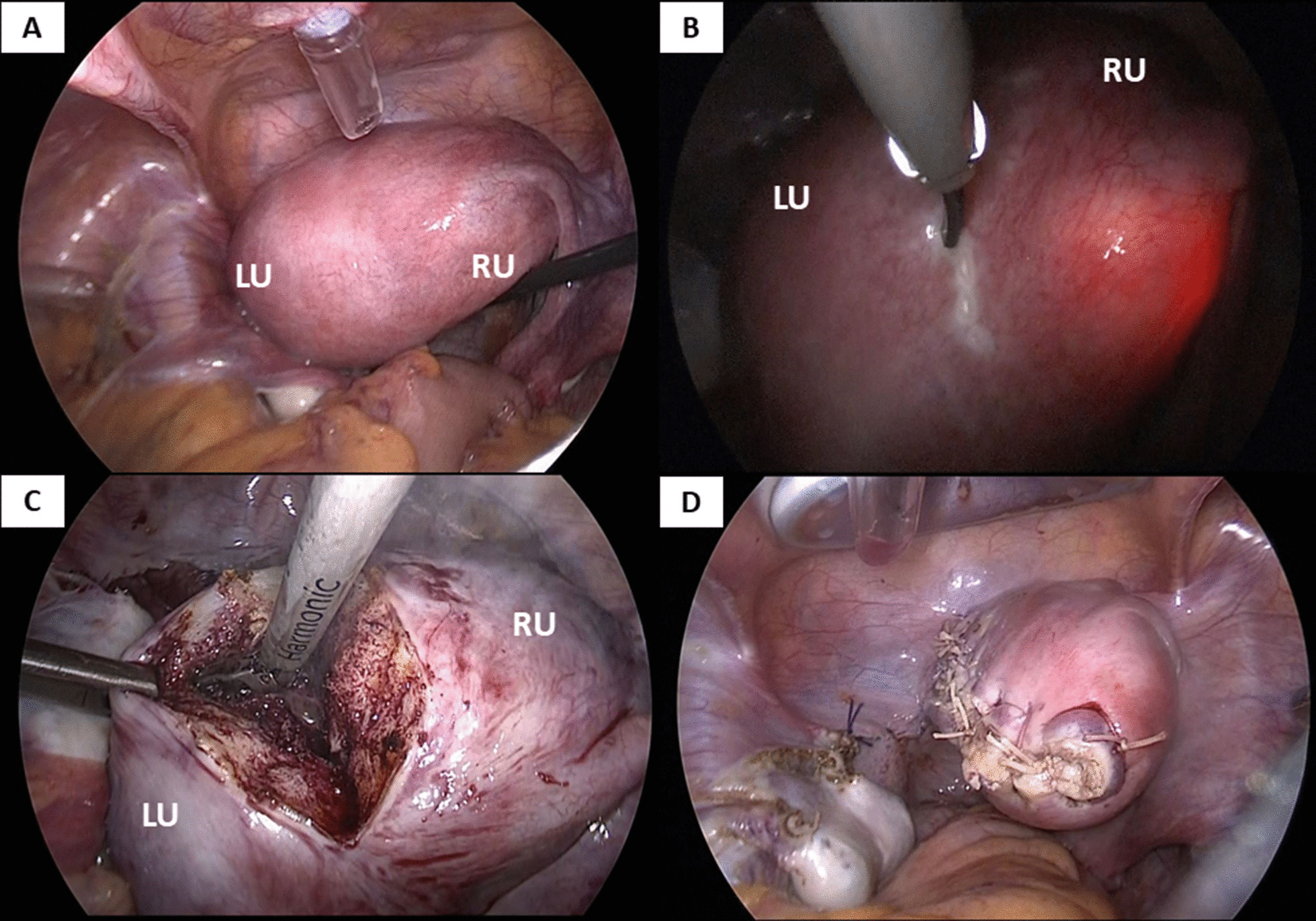
Laparoscopic intraoperative findings. **a** Intra-abdominal findings show slightly enlarged uterine corpus on the left side with normal bilateral adnexa. The uterine fundus is slightly concave, but not divided into two horns. **b** Hysteroscopy is inserted into the right uterus whose intra-cavity is illuminated and observed from the intra-abdomen by laparoscopy to mark the incision line. Left uterine cavity is not illuminated after its obliteration by the asymmetric uterine septum. **c** An incision is made longitudinally along the border between the hemi-uterus with the left blind cavity and the right unicornuate uterus in order to resect the left uterus with hematometra. **d** Final laparoscopic vision after resection of the left uterus with hematometra, left salpingectomy, and uteroplasty. *US* Uterine strand, *RU* Rudimentary uterus

Intra-abdominal findings showed a slightly enlarged uterine corpus on the left side due to the hematometra, but confirmed normal bilateral adnexa. The uterine fundus was slightly concave, but not divided into two horns (Fig. 2a). At this point, Robert’s uterus could not have been diagnosed because we had not noticed this anomaly. Had Robert’s uterus been diagnosed, hysteroscopic septal resection would have been performed, but laparoscopic resection of a functioning non-communicating left rudimentary horn and salpingectomy were performed instead. A hysteroscope was inserted into the right uterus, and only the right cavity was illuminated as the left cavity was obliterated due to an asymmetric uterine septum. The incision line of the uterus was determined by the hysteroscopic illumination of the uterine cavity (Fig. 2b). An incision was made longitudinally along the border between the hemi-uterus with the left blind cavity and the right unicornuate uterus, which was followed by the resection of the left uterus with hematometra (Fig. 2c). The muscular layer of the incision was then sutured, and uteroplasty was performed (Fig. 2d). Histopathological findings of the resected uterus revealed adenomyosis with a slightly atrophic endometrium.

**Fig. 3:**
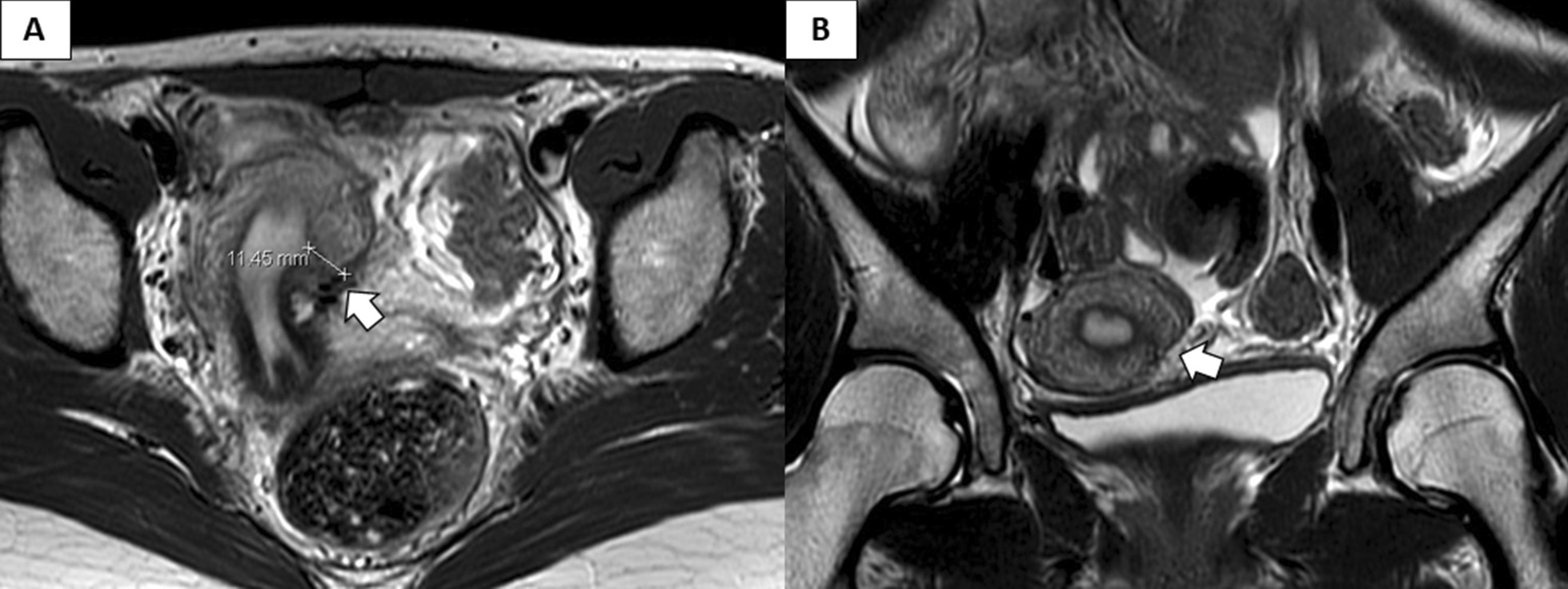
The findings of a follow-up MRI scan 3 years after the surgery. A uterus with a thick muscular layer (white arrow) was observed via the axial (**a**) and coronal (**b**) planes, and presented with a normal uterine appearance

The postoperative course was uneventful, and periodic menstruation started one month after the surgery; the severe lower abdominal pain was significantly relieved after the operation. After 3 years, a thick muscular layer, which appeared to be normal, was observed on the uterus during a follow-up MRI scan (Fig. 3).

## Discussion and conclusions

Robert’s uterus is a rare congenital Mullerian anomaly, characterized by an asymmetric septate uterus with a uterine septum dividing the uterine cavity into two distinct cavities: (1) a blind hemi-cavity and (2) a contralateral non-obstructing unicornuate uterine cavity that is typically connected to the cervix, with or without the small external indentation of a normal uterine fundus [2–4]. One blind hemi-cavity with a functional endometrium causes menstrual retention, leading to unilateral hematometra, hematosalpinx, and endometriosis. These phenomena are associated with the severity of abdominal pain and dysmenorrhea reported by the patient. The three main clinical features of Robert’s uterus are as follows: (1) a large hematometra in the blind hemi-cavity and acute pelvic pain, (2) an inactive blind hemi-cavity without hematometra and recurrent miscarriages, and (3) a small hematometra in the blind hemi-cavity [3].

Robert’s uterus is an exceptionally rare variant of the complete septate uterus because of its asymmetry. Furthermore, the classification of Robert’s uterus remains debatable as its embryological pathogenesis is still unclear. The European Society of Human Reproduction and Embryology–European Society for Gynecological Endoscopy classification system describes Robert’s uterus as a rare anomaly involving a complete septate uterus with unilateral cervical aplasia (class U2bC3V0) [5], without explaining the actual cause of the unilateral cervical aplasia. Therefore, Robert’s uterus is considered as class Vb under the American Society of Reproductive Medicine classification based on the notion that unilateral cervical aplasia may indicate the segmental agenesis of the isthmus without reabsorption of the septum between the upper regions of the Mullerian ducts [6].

Robert’s uterus is difficult to diagnose pre-operatively [4, 7–12], especially because abdominal pain is also caused by the more common endometriomas and hematosalpinx, resulting in its misdiagnosis. US and MRI are the modalities used for diagnosing Robert’s uterus with corresponding hysterosalpingography, hysteroscopy, and laparoscopy becoming useful, especially in cases where infertility is an issue. Moreover, the differential diagnosis of  a unicornuate uterus with a non-communicating rudimentary horn and hematometra should also be carefully considered as a more radical hemi-hysterectomy is the preferred method of treatment for this particular uterine anomaly [4, 13]. Provided that the sensitivity of US in diagnosing Robert’s uterus is not high, the evaluation of the external fundal contour becomes the key in differentiating between septate and bicornuate uteri. The normal uterine fundus is usually convex, but may sometimes be flat or slightly concave with a < 10-mm concavity between the right and left horns. However, the outer fundal contour of a bicornuate uterus presents with a larger or wider concavity at > 10 mm [3, 4]. Considering how rare Robert’s uterus is as well as the limitations of relevant imaging and standards, preoperative diagnosis is often wrong and the condition tends to be misdiagnosed as uterine adnexal diseases or appendicitis with right lower abdominal pain, leading to unsuccessful surgeries and treatment plans [4, 7–12]. Additionally, Robert’s uterus may be misdiagnosed as another uterine malformation disease, so uteroplasty may be performed incorrectly. In our case, MRI and intra-abdominal findings revealed a slightly concave uterine fundus, which we misdiagnosed as a unicornuate uterus with a non-communicating rudimentary horn and hematometra because we had no knowledge of this disease at that time. Consequently, we performed laparoscopic resection of the non-communicating rudimentary uterus (i.e., hemiuterus in this case) with ipsilateral salpingectomy to prevent future ectopic pregnancy and rupture of the pregnant horn [14, 15]. Importantly, to accurately diagnose and differentiate this entity from other similar uterine malformations, knowledge of this rare disease and the various diagnostic approaches such as MRI, US, hysteroscopy, and laparoscopy are essential. There is a need for a more tailored fit approach in evaluating uterine anomalies, which considers the age of the patient, presence of concurrent adenomyosis or endometriosis, and the patient’s desire for preserving fertility.

Clinical management of Robert’s uterus is not fully established as it has been reported in the literature mainly as case reports. Its treatment goals include drainage of the hematometra and prevention of its recurrence. Recurrence of hematometra can be prevented via complete excision of the obstructed cavity with preservation of the normal cavity, or via unification of both uterine cavities by incising the septum. Previously, the most common treatments for Robert’s uterus were laparoscopic or laparotomic resection of the hemi-uterus, or endometrectomy of the blind cavity [14, 16–19]. However, hysteroscopic metroplasty with US and laparoscopic guidance has recently become the first choice of treatment owing to its good outcomes due to its relative safety and low invasiveness. This approach can normalize uterine morphology and function, which can open up possibilities for improving the uterine cavity for better reproductive outcomes in the future [2, 3, 5–8]. Although laparoscopy has the advantage of treating superficial endometriosis and hematosalpinx, which occurs in 40 % of the patients with this anomaly [16], three-dimensional US may be an effective tool for the complex pre- and post-operative management of Robert’s uterus [2, 3, 6] as it provides an adequate understanding of the internal and external uterine structure and is considered an accurate method for diagnosing and classifying congenital uterine anomalies [20]. Moreover, US is well-tolerated, economically favorable, and easily available in routine clinical practice. Hysteroscopic metroplasty with transrectal US guidance may also be used as alternative approaches, which are safe for patients and reduce the need for more invasive procedures such as laparoscopy and laparotomy; primarily because the resolution of transrectal US is superior to that of transabdominal US [6].

Regarding fertility in patients with Robert’s uterus, recurrent pregnancy loss and infertility are the main clinical problems. Case reports of post-operative childbirth are limited [7, 9, 13, 21, 22], and the effect on pregnancy remains unclear. There are also a few reports of a rare complication of Robert’s uterus manifesting with pregnancy in a blind cavity, resulting in stillbirth associated with transperitoneal migration of sperms to the contralateral tube [23, 24]. Therefore, more long-term observations are necessary for the assessment of the reproductive outcomes of Robert’s uterus and its management in women.

Although there are other reports of excision of the hemi-uterus for a misdiagnosed Robert’s uterus [19], we could not correctly manage the presented case by conservative hysteroscopic unification. Due to the misdiagnosis and the fact that we were unaware of the Robert’s uterus anomaly, we inadvertently removed a portion of her uterus. Consequently, the patient’s symptoms were relieved, and the treatment outcome was tentatively considered satisfactory. Although a follow-up MRI scan revealed a normal uterine appearance, our surgical procedure may have left the lateral uterine wall weak. Considering fertility, the surgery may lead to more invasive and disadvantageous surgical treatment in future pregnancy as we have removed a significant portion of her uterus, making her cavity smaller, and much like a myomectomy, leaving behind a fragile uterine wall. Due to resection of the hemi-uterus, the probability of conception is expected to decrease and the risk of uterine rupture to increase in our patient. Therefore, her future pregnancies will require extremely close monitoring with caesarian delivery also becoming highly recommended for her future childbirths. Accordingly, we have counseled her that resection of the hemi-uterus may (1) compromise a successful full-term delivery, (2) increase her risk for uterine rupture in future pregnancy, and (3) require cesarean deliveries for any future childbirths.

The early diagnosis of Robert’s uterus remains challenging as the condition can be easily misdiagnosed or even left unnoticed. Currently, the existing studies on Robert’s uterus are all case reports, mostly involving intra-operative diagnoses. Consequently, there are still no guidelines for the early diagnosis of Robert’s uterus. To avoid misdiagnosis and inappropriate management, pediatricians, gynecologists, and surgeons should therefore be aware of this unique Mullerian anomaly and its proper management. In young women, especially those who suffer from severe dysmenorrhea, even if menstrual cycles and the appearance of the uterus are normal, the possibility of uterine malformation should always be considered.

In conclusion, we misdiagnosed Robert’s uterus without adequate knowledge of this disease and its management, resulting in the need for more invasive and inappropriate surgical treatment. The preoperative diagnosis of Robert’s uterus is difficult because of its rarity. Despite how challenging it is, its early and accurate diagnosis is crucial for the adequate planning of appropriate surgical intervention and management towards maintaining the quality of life and ensuring safety in future pregnancies.

## Data Availability

The data used during this study are available from the corresponding author on reasonable request.

## Abbreviations

US: Ultrasonoraphy
MRI: Magnetic resonance imaging
